# Targeting G_1_/S phase cell-cycle genomic alterations and accompanying co-alterations with individualized CDK4/6 inhibitor–based regimens

**DOI:** 10.1172/jci.insight.142547

**Published:** 2021-01-11

**Authors:** Shumei Kato, Ryosuke Okamura, Jacob J. Adashek, Noor Khalid, Suzanna Lee, Van Nguyen, Jason K. Sicklick, Razelle Kurzrock

**Affiliations:** 1 Center for Personalized Cancer Therapy and Division of Hematology and Oncology, Department of Medicine, Moores Cancer Center at UC San Diego Health, La Jolla, California, USA.; 2 Department of Internal Medicine, University of South Florida, H. Lee Moffitt Cancer Center & Research Institute, Tampa, Florida, USA.; 3 Center for Personalized Cancer Therapy and Division of Surgical Oncology, Department of Surgery, Moores Cancer Center at UC San Diego Health, La Jolla, California, USA.

**Keywords:** Oncology, Cell cycle, Molecular biology

## Abstract

**BACKGROUND:**

Although CDK4/6 inhibitors are an established treatment for hormone receptor–positive, HER2-negative metastatic breast cancers, their benefit in other malignancies remains limited.

**METHODS:**

We investigated factors associated with clinical outcomes from CDK4/6 inhibitor–based therapy among patients with G_1_/S phase cell-cycle alterations (*CDK4/6* amplifications, *CCND1/2/3* amplifications, or *CDKN2A*/*B* alterations).

**RESULTS:**

Overall, 2457 patients with diverse solid tumors that underwent clinical-grade, next-generation sequencing (182–465 genes) and therapy outcome of (non–breast cancer) patients treated with matched CDK4/6 inhibitors were analyzed. G_1_/S phase cell-cycle alterations occurred in 20.6% (507 of 2457) of patients; 99% of those patients (*n* = 501) harbored ≥1 characterized co-alteration (median, 4; range, 0–24). In 40 patients with G_1_/S phase cell-cycle alterations given CDK4/6 inhibitors as part of their regimen, significantly longer median progression-free survival (PFS) was observed when CDK4/6 inhibitor–based therapies matched a larger proportion of tumor alterations, often because CDK4/6 inhibitors were administered together with other drugs that were matched to genomic co-alterations, hence achieving a high matching score (high vs. low [≥50% vs. <50%] matching score, PFS, 6.2 vs. 2.0 months, *P* < 0.001 [*n* = 40] [multivariate]) and higher rate of stable disease ≥6 months or an objective response (57% vs. 21%, *P* = 0.048).

**CONCLUSION:**

In summary, in cell-cycle–altered cancers, matched *CDK4/6* inhibitors, as part of an individualized regimen targeting a majority of genomic alterations, was independently associated with longer PFS.

**TRIAL REGISTRATION:**

ClinicalTrials.gov NCT02478931.

**FUNDING:**

Joan and Irwin Jacobs Fund, National Cancer Institute (P30 CA023100, R01 CA226803), and the FDA (R01 FD006334).

## Introduction

The cell cycle is tightly regulated by various checkpoints, which are populated by signaling molecules that need to be at threshold levels and appropriately phosphorylated by various kinases (1). Certain cancers have the ability to hijack various players within these cycle cascades, triggering uncontrolled growth and cell division (2). Specifically, cyclin-dependent kinases 4 and 6 (CDK4/6) require cyclin D1 to function and phosphorylate the retinoblastoma (RB) protein, which inactivates this tumor suppressor, allowing for the progression of cell cycle during the G_1_/S phase. Without cyclin D1 or appropriately functioning CDK4/6 enzymes, the cells will not adequately divide and proliferate. This cell-cycle feedback loop permits regulated growth and assures that cells only divide when necessary. However, several genomic alterations inappropriately fuel cell-cycle progression. Well-defined cyclin-related mechanisms that drive tumorigenesis include increases in expression/amplification of CDK4/6, upregulation of cyclin D, or deleterious alterations in the *Rb*, *CCNE1*, *CDKN2A*, or *CDKN2B* genes (3–5). The aforementioned G_1_/S phase cell-cycle modulator alterations exist in anywhere from 9.5% to 73.8% of a variety of tumor types, making this pathway an attractive therapeutic target (6).

There are currently 3 CDK4/6 inhibitors — palbociclib, ribociclib, and abemaciclib — that are FDA approved for the treatment of hormone receptor–positive (HR-positive), HER2-negative metastatic breast cancer in conjunction with an aromatase inhibitor (7–10). While these agents result in improved progression-free survival (PFS) and overall survival (OS) in this patient population, there remains no clear biomarker that predicts response to CDK4/6 inhibitors (11–13). Theoretically, amplification of *CDK4/6* and cyclin D1, D2, and/or D3 (*CCND1/2/3*) and alterations in *CDKN2A*/*B* are putative markers to predict the response from CDK4/6 inhibitors; however, there have mixed reports in this regard. For instance, in the American Society of Clinical Oncology’s TAPUR study, participants with *CDKN2A* alterations (expected to increase CDK4/6 expression) or *CDK4* amplifications were assigned to receive palbociclib. Patients with head and neck cancer, soft tissue sarcoma, and bronchus/lung cancers did demonstrate benefit and continued on to the second portion of the trial as part of Simon’s optimal 2-stage design (14). However, patients with pancreatic and gallbladder cancers with *CDKN2A* alterations did not derive significant benefit from CDK4/6 inhibition. The discrepancy in findings between tumor histologies confounds the ability to identify a biomarker of responsiveness. Furthermore, no cassette of markers has proved important in patients with breast cancer treated with CDK4/6 inhibitors (15). It is therefore still unclear, despite the pharmacologically driven properties of these agents supporting their effect on the G_1_/S phase cell-cycle pathway, how to best ascertain in advance if there is a subset of non–breast cancer patients who may respond to CDK4/6 inhibitors,

One hypothesis for why certain G_1_/S phase cell-cycle–associated genes have not been reliable markers to predict sensitivity to CDK4/6 inhibitors (11, 16) relates to the frequent finding of important genomic co-alterations (2). On average, patients with metastatic cancer have approximately 2–5 deleterious genomic alterations when assessed with a fixed panel derived from next-generation sequencing (NGS) (17–19). Although targeting the cell-cycle pathway may be appealing, it may also be less rewarding than anticipated due to this phenomenon. Indeed, although certain drivers, such as *EGFR* or *ALK* or *NTRK* aberrations, may be effectively targeted by matched monotherapy, not all patients respond and resistance often develops (20–23). It is plausible, therefore, that, even in these cases, primary or secondary resistance could be driven by co-alterations or driver feedback loops. For instance, in colorectal cancer with *BRAF* mutations, BRAF inhibitors alone are ineffective. Meanwhile, the BRAF inhibitor encorafenib, together with the EGFR antibody cetuximab, targets both BRAF and the feedback EGFR driver pathway; this efficacious combination was recently approved by the FDA (24). Indeed, targeting one specific signal in a complicated network of genomic drivers may be ineffective (25), and recent studies demonstrate that the greater the proportion of signals targeted, the better the outcome (26–28).

Herein, we used NGS to interrogate the complex genomic landscape of 2457 patients with diverse cancers, of whom 507 patients harbored specific, potentially sensitizing G_1_/S phase cell-cycle (*CDK4/6*, *CCND1/2/3*, or *CDKN2A*/*B*) gene alterations. In a subset of patients with cancer (not breast cancer) with sensitizing cell-cycle gene alterations treated with CDK4/6 inhibitors, we show examples of responders. Additionally, we show that, overall, there was a significantly improved PFS and higher rate of stable disease ≥6 months or having a response observed when a majority of genomic alterations/co-alterations were targeted, as compared with administration of matched CDK4/6 inhibitors alone in the face of complex molecular portfolios.

## Results

### Characteristics of patients with alterations in the potentially sensitizing G_1_/S phase cell-cycle signaling pathway (CDK4/6 amplifications, CCND1/2/3 amplifications, or CDKN2A/B alterations).

**Figure 1:**
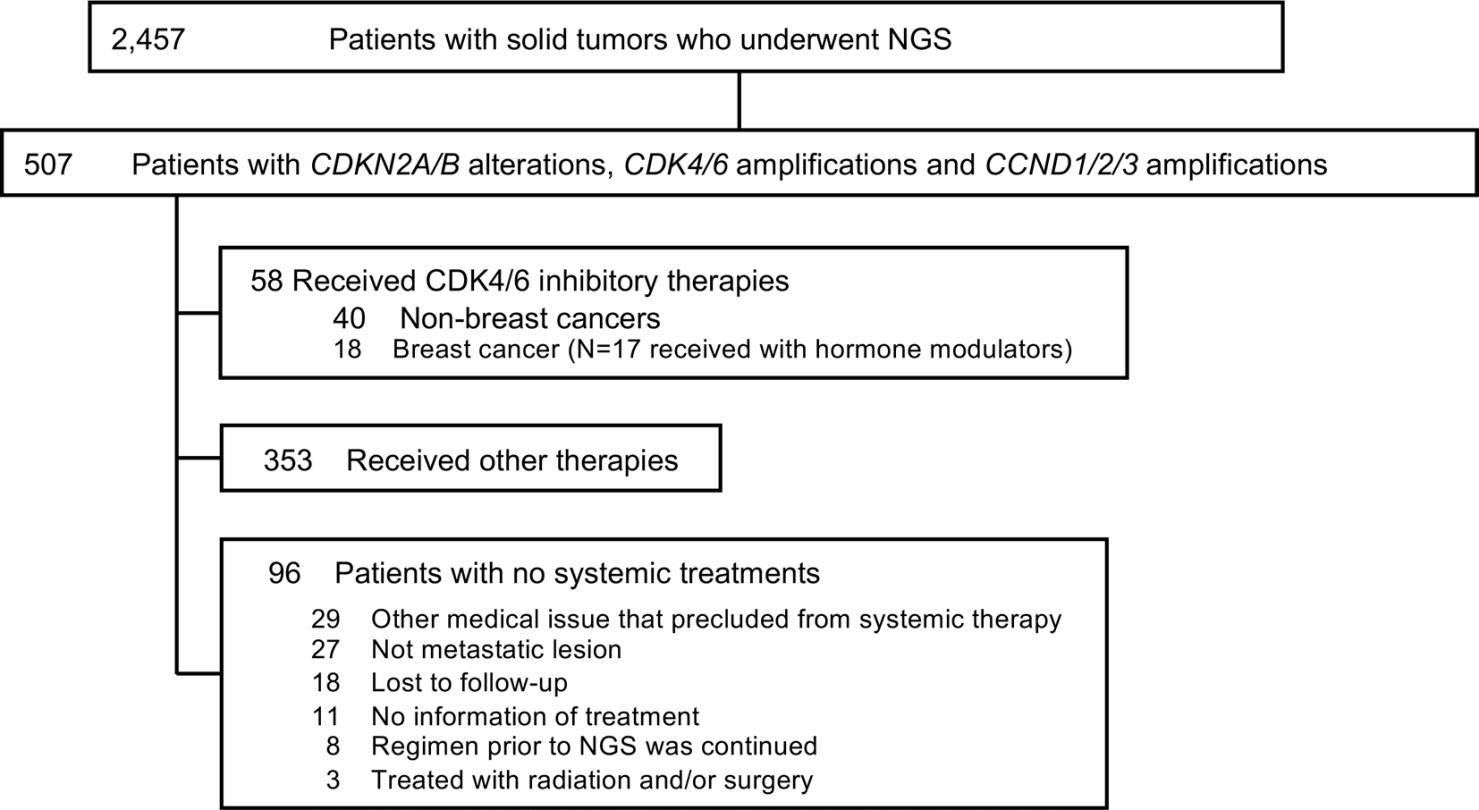
Consort diagram of patients with alterations in the G_1_/S phase cell-cycle signaling pathway (*n* = 507).

Among 2457 patients with diverse solid tumors, 507 patients (21%) had at least 1 characterized genomic alteration in sensitizing G_1_/S phase cell-cycle signaling genes — *CDK4/6*, *CCND1/2/3*, or *CDKN2A*/B — on tissue NGS (Figure 1). Among the 507 patients, the most common diagnosis was brain tumors (16%, *n* = 83), non–small cell lung cancers (15%, *n* = 77), and skin cancers, including melanoma (13%, *n* = 67). Among the G_1_/S phase cell-cycle alterations of interest, *CDKN2A*/B alterations (71%, *n* = 359) were the most commonly observed in this series, followed by *CCND1* amplification (15%, *n* = 75) and *CDK4* amplification (12%, *n* = 61) (Table 1).

### Most patients with alterations in G_1_/S phase cell-cycle signaling pathway had genomic co-alterations.

**Figure 2:**
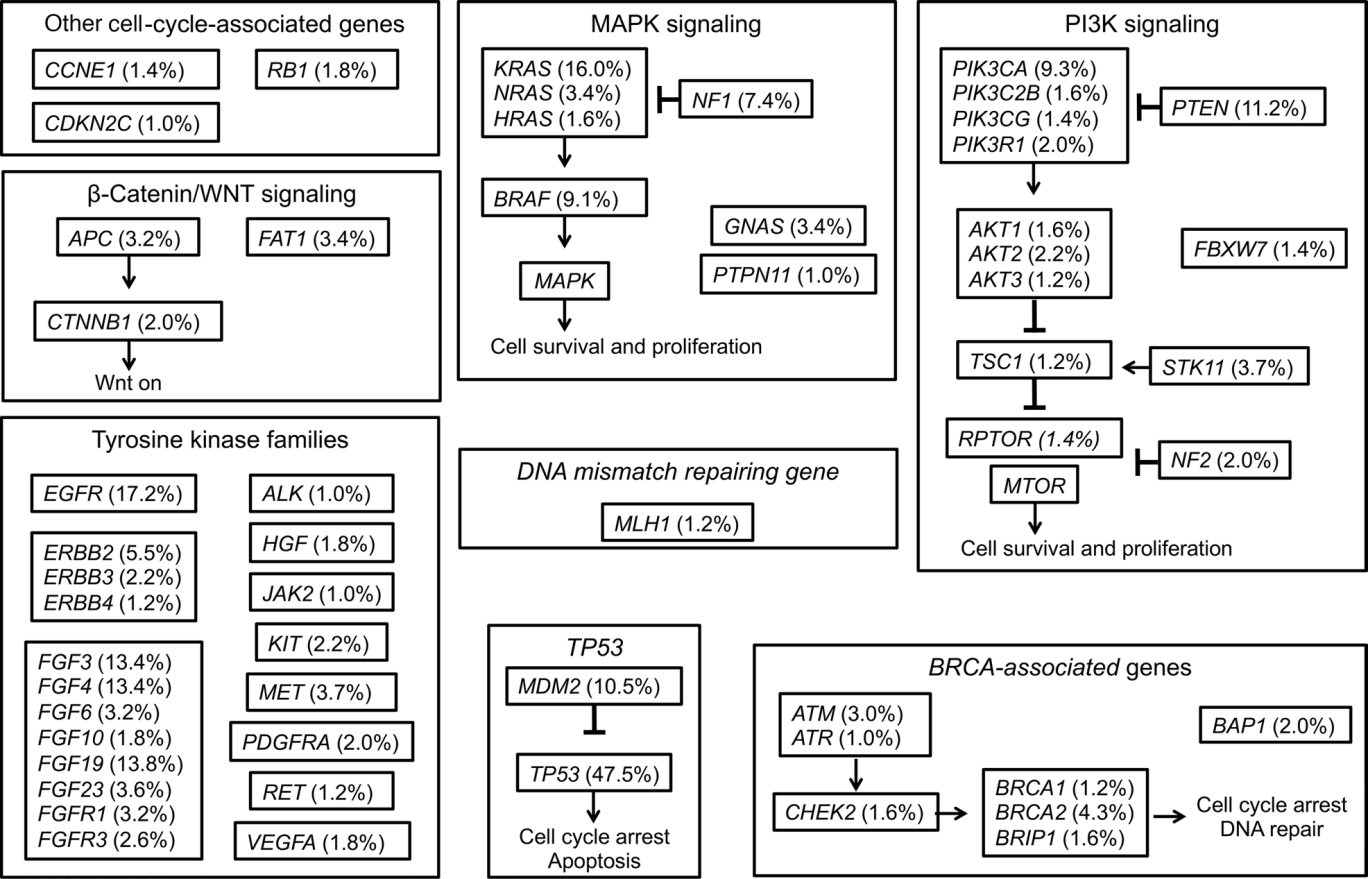
Summary of co-alterations observed in tumors harboring *CDK4/6* amplifications, *CCND1/2/3* amplifications, or *CDKN2A/B* alterations (*n* = 507). Among 507 patients with diverse tumors harboring *CDK4/6* amplifications, *CCND1/2/3* amplifications, or *CDKN2A/B* alterations, most patients (99%, *n* = 501) had ≥1 characterized co-alteration (median, 4; range, 0–24) in tissue NGS. The most common co-alterations were in *TP53* (approximately 48% of patients, *n* = 241), *EGFR* (17% of patients, *n* = 87), *TERT* (16% of patients, *n* = 82), and *KRAS* genes (16% of patients, *n* = 81). Genomic alterations with frequency of ≥1.0% were included.

Among 507 patients with diverse tumors harboring *CDK4/6* amplifications, *CCND1/2/3* amplifications, or *CDKN2A/B* alterations, 99% (*n* = 501) had at least 1 deleterious co-alteration (median, 4 co-alterations [excludes the cyclin alteration]; range, 0–24) in tissue NGS and the remaining 6 patients whose tumors did not have a co-alteration only had a *CDKN2A/B* alteration. The most common co-alterations were seen in the *TP53* (48% of the 507 patients, *n* = 241), *EGFR* (17%, *n* = 87), *TERT* (16%, *n* = 82), and *KRAS* (16%, *n* = 81) genes (Figure 2). Co-alterations in cell-cycle resistant genes (*RB* and *CCNE1*) were rare (each occurring in less that 2% of cases).

### In patients with cell-cycle–altered tumors, CDK4/6 inhibitor–based therapy with high matching score was associated with significantly longer PFS and a higher rate of clinical benefit (stable disease ≥6 months or objective response).

Among 507 patients with *CDK4/6* amplifications, *CCND1/2/3* amplifications, or *CDKN2A/B* alterations, 40 patients with diverse cancers (excluding patients with breast cancer) were treated with CDK4/6 inhibitor–containing regimens and evaluated for PFS (Figure 1 and Supplemental Table 1; supplemental material available online with this article; https://doi.org/10.1172/jci.insight.142547DS1). (The breast cancer cohort was excluded from the analysis because the combination of CDK4/6 inhibitors with antihormone agents is already FDA approved for patients with breast cancer [ref. 29]). None of the treated patients had a coexisting cell-cycle gene alteration in *RB* or *CCNE1*.

**Figure 3:**
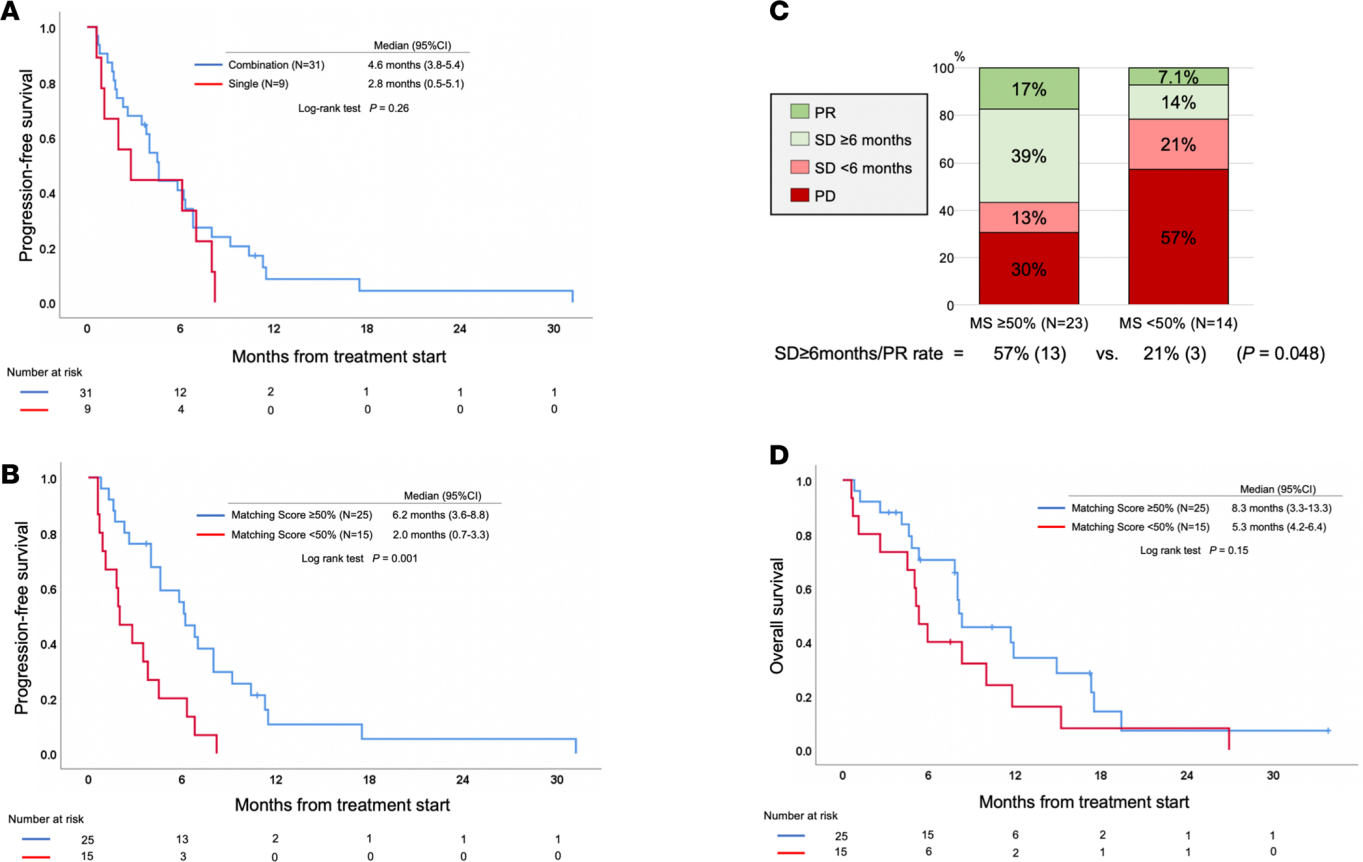
Progression-free survival among patients with alterations in *CCND1/2/3*, *CDK4/6*, and/or *CDKN2A/B* G_1_/S phase cell-cycle genes, who received CDK4/6 inhibitor–based therapy (*n* = 40). (**A**) Progression-free survival (PFS) comparison between patients who received CDK4/6 inhibitor–based therapy as part of the combination therapies (*n* = 31) and patients who received CDK4/6 inhibitor as a single agent (*n* = 9). Among patients with diverse cancers (*n* = 40) who received CDK4/6 inhibitor–based therapy, there was no significant difference in PFS between patients who received combination therapy and those who received single agents (combination vs. single agent, 4.6 vs. 2.8 months, *P* = 0.26). (**B**) PFS among patients who received CDK4/6 inhibitor–based therapy with a matching score of ≥50% (*n* = 25) versus those with a matching score of <50% (*n* = 15). Among patients with diverse cancers (*n* = 40) who received CDK4/6 inhibitor–based therapy, patients who were treated with a combination of agents with higher matching scores had significantly longer PFS (median PFS for matching score ≥50% vs. <50%, 6.2 vs. 2.0 months, *P* = 0.001). (**C**) Response to CDK4/6 inhibitor–based therapies among patients with CCND1/2/3, CDK4/6, and/or CDKN2A/B G_1_/S phase cell-cycle gene alterations. Comparison between patients who received CDK4/6 inhibitor–based therapy with matching score of ≥50% (*n* = 23) and patients with matching score of <50% (*n* = 14). There was a significant difference in achieving stable disease ≥6 months/partial response among patients who received therapy with matching score of ≥50% as compared with those with a matching score of <50 (57% vs. 21%, *P* = 0.048). (Among 40 patients treated with matched CDK4/6 inhibitor–based therapies, 37 patients were assessable for response.) (**D**) Overall survival (OS) comparison (*n* = 40) between patients who received CDK4/6 inhibitor–based therapy with matching score of ≥50% (*n* = 25) and patients with matching score of <50% (*n* = 15). Among patients who received CDK4/6 inhibitor–based therapy (*n* = 40), there was no significant difference in OS between those with a matching score of ≥50% vs. <50% (median OS between matching score ≥ 50% vs. < 50%, 8.3 vs. 5.3 months; *P* = 0.15). Reverse Kaplan-Meier calculation for **A**, **B**, and **D** revealed no difference between groups, indicating that the median follow-up between groups was similar. MS, matching score; PD, progressive disease; PR, partial response; SD, stable disease.

Among those 40 patients with diverse cancers, PFS was not associated with age, sex, types of cancer, line of therapy, or treatment regimen in univariate analysis (Table 2 and Figure 3A). Among patients with *CDKN2A/B* alterations, PFS was worse, but the difference was not significant (median PFS between *CDKN2A/B* alteration vs. not, 4.0 vs. 6.8 months; *P* = 0.10 [univariate]). PFS was significantly longer among patients with Eastern Cooperative Oncology Group Performance Status (ECOG PS) of 0–1 (median PFS between ECOG PS 0–1 vs. 2–3, 6.1 vs. 1.6 months; *P* = 0.04) and in patients who had a higher matching score (i.e., matching score roughly equivalent to the number of alterations targeted divided by total number of deleterious alterations) (median PFS between matching score ≥50% vs. <50%, 6.2 vs. 2.0 months; *P* = 0.001 [univariate]) (Table 2 and Figure 3B).

After the multivariate analysis, *CDKN2A/B* alteration remained a factor independently associated with poor PFS (HR, 2.76; 95% CI, 1.10–6.93; *P* = 0.03) and high matching score was an independent factor for longer PFS (≥ 50% vs. <50%) (HR, 0.24; 95% CI, 0.11–0.51; *P* < 0.001) (Table 2).

In line with the favorable longer PFS seen, patients with a matching score of ≥50% achieved stable disease for ≥6 months or a stable objective response rate at higher rates (matching score of ≥ 50% vs. < 50%, 57% vs. 21%, *P* = 0.048) (Figure 3C). Improved PFS with a higher matching score also translated into numerically longer median OS, which, however, was not statistically significant (median OS between matching score ≥50% vs. <50%, 8.3 vs. 5.3 months; *P* = 0.15 [univariate]) (Figure 3D). Similar clinical outcomes were observed among the 33 patients who were managed with regimens that did not contain immunotherapy (matching score ≥50% vs. <50%, PFS, 8.8 vs. 3.2 months [*P* = 0.001], OS, 13.0 vs. 8.0 months [*P* = 0.08] [univariate]) (Supplemental Table 1).

### Examples of responding patients treated with CDK4/6 inhibitory therapy.

**Figure 4:**
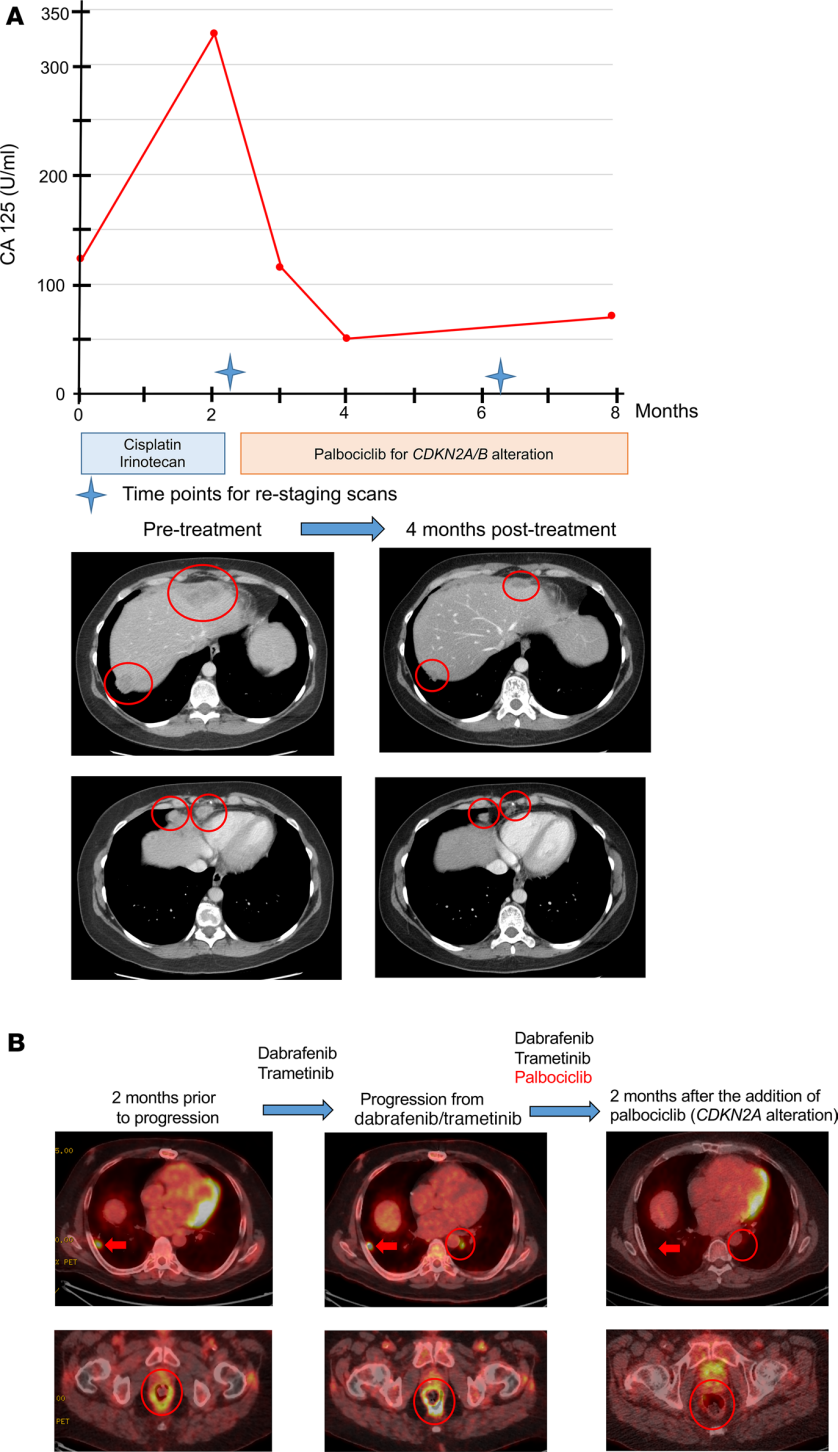
Examples of responders treated with CDK4/6 inhibitory therapy. (**A**) Case 1 (patient ID 269): Forty-three-year-old woman with metastatic high-grade ovarian carcinoma with neuroendocrine features that harbored CDKN2A/B alteration without any genomic co-alterations on the NGS panel of 315 genes and 2 lines of prior therapy demonstrated partial response with single-agent palbociclib lasting 8 months. NGS of tumor showed a single alteration in *CDKN2A/B*, for which the patient was started on palbociclib. Restaging scan with CT overall showed 30% regression, indicating partial response at the 4-month time point (response by RECIST 1.1). Along with the radiographic response, reduction of tumor marker, CA 125 was seen (CA 125: 328 U/ml down to 50 U/ml [reference range 0–34 U/ml]). (**B**) Case 2 (patient ID 501): Sixty-eight-year-old man with metastatic gastrointestinal stromal tumor (GIST) with alterations in *BRAF* V600E, *CDKN2A* p16INK4a splice site 150+1G > A and *LRP1B* deletion exon 23 presented after progressing treatment with dabrafenib (BRAF inhibitor) and trametinib (MEK inhibitor) based on underlying *BRAF* V600E mutation (30). Addition of palbociclib led to partial response lasting 11.3 months. Although progression was seen with a new pulmonary nodule and worsening rectal lesion (left to middle, circle), one of the right lower lung masses appeared to be stable (left to middle, arrow), and thus the decision was made to continue on dabrafenib/trametinib and to add palbociclib based on additional alteration in *CDKN2A*. Two months after the addition of palbociclib, restaging scan with PET/CT scan showed resolution of ^18^F-fluorodeoxyglucose–avid lung nodules as well as improvement in rectal lesion (middle to right). PFS was 11.3 months without significant toxicities.

Case 1 (patient ID 269) is a 43-year-old woman with metastatic high-grade ovarian carcinoma with neuroendocrine features and 2 prior lines of therapy. The patient’s tumor harbored a sole alteration in *CDKN2A/B* and demonstrated a response with single-agent palbociclib (30% regression; partial response by RECIST 1.1; tumor marker, CA 125, 328 [baseline] down to 50 U/ml [reference range, 0–34 U/ml], PFS, 8.0 months) (Figure 4A and Supplemental Table 1).

Case 2 (patient ID 501) is a 68-year-old man with metastatic gastrointestinal stromal tumor with *BRAF* V600E and *CDKN2A* alterations (30), who presented after the tumor progressed on BRAF/MEK-targeted therapy. Addition of palbociclib led to resolution of ^18^F-fluorodeoxyglucose–avid diseases per PET/CT scan and PFS of 11.3 months without significant toxicities (Figure 4B and Supplemental Table 1).

## Discussion

The cell cycle allows normal cellular growth and proliferation and is highly regulated by a series of cyclin molecules and their dependent constellation of kinases, whose signals must be integrated to determine if it is appropriate for cells to divide. The activity of these kinases relies on the production of their cognate cyclin partners, represented by the D-type cyclins CCND1/2/3. In parallel, CDK4/6 kinases are also regulated by phosphorylation events and the presence of physiological kinase inhibitory proteins. These inhibitors are encoded by the *CDKN2* gene family — *CDKN2A* and *CDKN2B*, which yield selective CDK inhibitors for CDK4/6 (e.g., p16INK4a and p15INK4b) (31). The dysregulation through genomic alteration of the aforementioned major players, including *CDK4/6*, *CCND1/2/3*, and *CDKN2A/B* genes, has been implicated in the pathogenesis of diverse malignancies. Currently, CDK4/6 inhibitors are approved for patients with HR-positive, HER2-negative metastatic breast cancer, as these inhibitors improve PFS and OS when given with hormone modulators (7–10). Even so, investigators have failed to identify a reliable biomarker for CDK4/6 inhibitors, despite attempts made in several studies of breast cancer (11–13, 15). Moreover, CDK4/6 inhibitors given as monotherapy matched to cognate alterations in a variety of cancers have mostly fared poorly (14, 15).

We hypothesized that the lack of association between *CDK4/6* amplifications, *CCND1/2/3* amplifications, and/or *CDKN2A/B* alterations and outcome after administration of CDK4/6 inhibitors may be due to intratumoral heterogeneity and complexity, resulting in a large proportion of metastatic tumors with cyclin alterations also carrying genomic co-alterations that differ from patient to patient. Consistent with our hypothesis, in our cohort of 507 patients with a variety of cancers harboring G_1_/S phase cell-cycle gene alterations (*CDK4/6* amplifications, *CCND1/2/3* amplifications, or *CDKN2A/B* alterations), 99% of patients (*n* = 501) had at least one genomic co-alteration (median, 4; range, 0–24). These alterations were heterogeneous and affected multiple oncogenic signaling pathways, including those regulated by mitogen-activated protein kinase, phosphoinositide 3-kinase, and β-catenin/Wnt; other kinase families and *BRCA*-associated genes were also affected (Figure 2).

The presence of coexisting disrupted oncogenic pathways could potentially lead to resistance to CDK4/6 inhibitors. Consistent with this notion, in the current study, even combination therapy that matched CDK4/6 inhibitors to *CDK4/6* amplifications, *CCND1/2/3* amplifications, or *CDKN2A/B* alterations, but without necessarily matching to genomic co-alterations, did not achieve a better clinical outcome when compared with patients who received matched CDK4/6 inhibitors alone (combination approach [implying matched CDK4/6 inhibitor and at least one other drug] vs. CDK4/6 inhibitor alone, PFS, 4.6 months vs. 2.8 months, *P* = 0.26) (Table 2). However, when the CDK4/6 inhibitor–based regimens were given together with customized additional drugs matched to genomic co-alterations in that patient’s tumor (resulting in a high matching score (≥50%), overall longer PFS was observed when compared with that of patients who were treated with a CDK4/6-matched regimen with a low matching score (<50%) (PFS 6.2 vs. 2.0 months, *P* < 0.001 [*P* values were calculated after multivariate analysis]) (Table 2). The clinical benefit rate (stable disease ≥6 months as well as a higher objective response rate) was also improved (57% vs. 21%; *P* = 0.048) (Figure 3C).

In the era of precision oncology, the majority of cancer clinical trials are aimed at a prespecified genomic target of interest, and many patients are being treated with single-matched drugs. Salutary effects have been observed by targeting certain genomic alterations, such as *NTRK*, *RET*, and *ALK* fusions or *BRAF* V600 as well as *EGFR* mutations (22, 32–35). However, resistance is inevitable and there is likely a limitation of benefit with single agents in the setting of genomically complex advanced cancers. To overcome these limitations, future directions for the development of cancer clinical trials may require a more flexible, individualized treatment strategy that is tailored to each patient’s tumor genomic profile. To this end, we have recently reported the outcome of the I-PREDICT and WINTHER trials, wherein we investigated personalized approaches based on genomic and/or transcriptomic profiling among patients with treatment-refractory solid tumors (26, 28). We demonstrated improvement in PFS and OS when targeting a larger fraction of identified molecular alterations, reflecting a high matching score, consistent with the observations in the current study. Further prospective trials with this tactic focused on patients with cell-cycle alterations are required.

There are several important limitations to the current report. First, the study has a small sample size. Second, while PFS and clinical benefit rate were improved with a greater degree of matching, survival changes did not reach statistical significance. A larger prospective trial that is controlled and randomized is needed, especially to mitigate the effect of confounders that may not be known despite the multivariate analysis. Third, molecular characteristics of tumors can have dynamic changes, especially with therapeutic pressure. Future studies may require serial profiling, such as with circulating tumor cell-free DNA analysis. Fourth, our study assessed only pathogenic somatic alterations, and not germ-line anomalies. Finally, this study included heterogeneous cancer diagnoses, and the number of patients in specific histologies was small (and patients were treated at various time points in their disease), which precluded the ability to interpret the results for specific disease types.

In conclusion, we have evaluated 2457 patients with diverse solid tumors and shown that potentially sensitizing G_1_/S phase cell-cycle molecular aberrations, such as *CDK4/6* amplifications, *CCND1/2/3* amplifications, and/or *CDKN2A/B* alterations, were observed in 507 patients (21%). Most participants with these alterations (99% of patients, 501 of 507) had at least one genomic co-alteration. Among patients with *CDK4/6* amplifications, *CCND1/2/3* amplifications, and/or *CDKN2A/B* abnormalities, adding additional drugs to the CDK4/6 inhibitor–based regimen without consideration of genomic co-alterations did not improve clinical outcome. However, significant improvement in PFS and in clinical benefit rate were observed when matched CDK4/6 inhibitors were given as part of a tailored regimen that affected a larger proportion of genomic alterations, with achievement of a high matching score. Because the genomic co-alterations differed from patient to patient, individualized combination therapies were often required. These results imply that, in the case of CDK4/6 inhibitors given to patients whose tumors harbor potentially sensitizing cyclin alterations, personalized consideration of important molecular co-alterations warrants further investigation as a direction for achieving benefit.

## Methods

### Study population.

Patients were generally matched after presentation to a Molecular Tumor Board (36–38). Some patients were navigated to prospective precision studies, such as I-PREDICT (28). A total of 2457 patients with solid tumors who underwent tissue NGS were analyzed from January 2013 to April 2018. All patients were at UCSD. Among them, 507 patients with genomic alterations in *CDK4/6*, *CCND1/2/3*, or *CDKN2A*/B were included for more in-depth assessment (*n* = 507) (Figure 1). These genes were chosen because they are potentially sensitizing to CDK4/6 inhibitors.

### Tissue NGS.

All tissue DNA analyses were performed by a clinical laboratory improvement amendments–certified lab, Foundation Medicine Inc., as per methods previously described in detail (39) (https://www.foundationmedicine.com), except for 2 patients, with analyses performed at UCSD NGS and HLIQ Oncology (182–465 cancer-related genes). Briefly, 50–200 ng genomic DNA was extracted and purified from the submitted FFPE tumor samples. DNA was adaptor ligated, and hybrid capture was performed for all coding exons of 182–406 cancer-related genes plus selected introns from 14–31 genes frequently rearranged in cancer (Illumina HiSeq platform). Sequencing was performed with an average sequencing depth of coverage of >250×, with >100× at >99% of exons. Somatic mutations were identified with >99% sensitivity and 99% specificity for base substitutions at >95% sensitivity for copy number alterations, and ≥5% mutant allele frequency. Gene amplification was reported at ≥8 copies above ploidy, with ≥6 copies considered equivocal (with the exception of *ERRB2*, for which ≥5 copies is considered equivocal amplification). Tumor-mutation burden was classified into 3 categories: low (<6 mutations/mb), intermediate (6–19 mutations/mb), and high (≥20 mutations/mb). Variants of unknown significance were excluded from all analyses.

### Molecular matching score.

The molecular matching score was developed in an attempt to assess the association between coverage of deleterious genomic alterations by targeted therapy that patients may have received based on these molecular alterations and the clinical outcome, as previously described (27, 28, 41, 42). The score is roughly equal to the total number of deleterious alterations affected divided by the total number of deleterious alterations in each patient. Under this system, the higher the molecular matching score, the better the match. See Supplemental Methods for further description. Matching scores were determined while blinded to outcome.

### Statistics.

Patient characteristics; prevalence of alterations in *CDK4/6*, *CCND1/2/3*, or *CDKN2A*/B; and genomic co-alterations were summarized by descriptive statistics such as Kaplan-Meier and Log-rank test were used (as stated below). Among 40 patients with cancer (but not breast cancer) who underwent CDK4/6 inhibitor–based therapies, we assessed PFS, which was defined as time between start of the treatment and disease progression confirmed by imaging or clinical findings. OS was defined as time between start of therapy until the last follow-up. Patients with ongoing therapy without progression at the last follow-up date were censored for PFS at that date. Patients alive at last follow-up were censored for OS. PFS and OS were assessed by the Kaplan-Meier method. Reverse Kaplan-Meier was also done to determine whether there were differences in median follow-up times between groups (40). Log-rank test and Cox regression analysis were used to compare subgroups of patients. All tests were 2 sided, and variables with *P* ≤ 0.1 were included for multivariate analysis. *P* ≤ 0.05 was considered significant. Statistical analyses were performed using SPSS version 24 software (IBM Corporation).

### Study approval.

All investigations in this study were approved by and analyzed according to the guidelines of Moores Cancer Center at UC San Diego Health Internal Review Board under the Profile-Related Evidence Determining Individualized Cancer Therapy study (PREDICT study, NCT02478931). Patients gave informed consent before participating in investigational therapies.

## Author contributions

SK, JJA, JKS, and RK drafted the manuscript; SK and RK designed the study; SK and RO analyzed the data; and RO, NK, SL, and VN collected the data. All authors read and approved the final manuscript.

## Supplementary Material

Supplemental data

## Figures and Tables

**Table 1:**
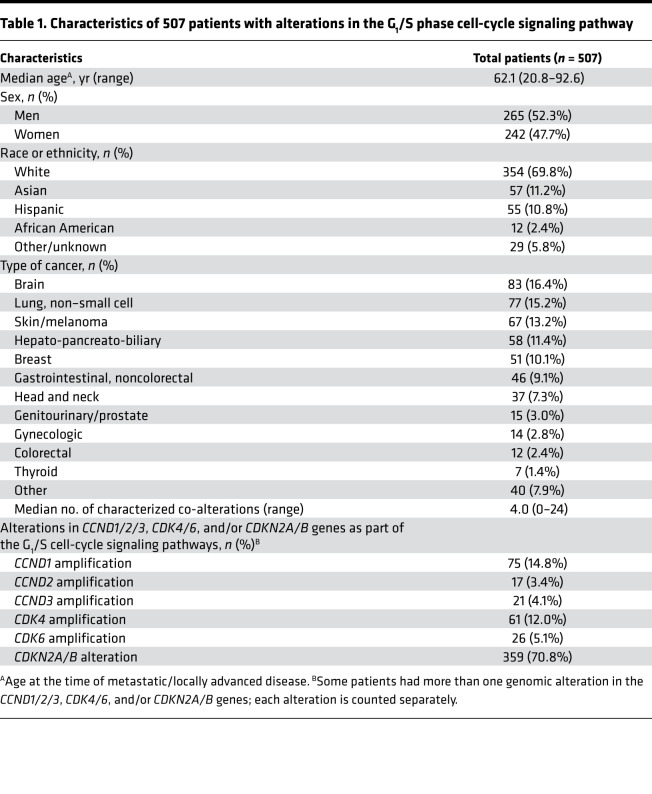
Characteristics of 507 patients with alterations in the G_1_/S phase cell-cycle signaling pathway

**Table 2:**
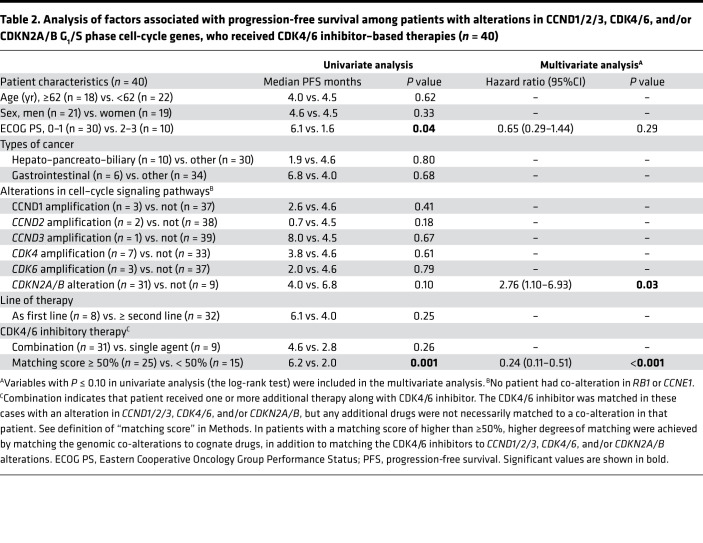
Analysis of factors associated with progression-free survival among patients with alterations in CCND1/2/3, CDK4/6, and/or CDKN2A/B G_1_/S phase cell-cycle genes, who received CDK4/6 inhibitor–based therapies (*n* = 40)

## Version 1 01/11/2021

Electronic publication
