# Supplementary material for: Targeting G1/S phase cell-cycle genomic alterations and accompanying co-alterations with individualized CDK4/6 inhibitor–based regimens
Source: JCI Insight. 2021 Jan 11;6(1):e142547. doi: 10.1172/jci.insight.142547 (PMC7821594; doi:10.1172/jci.insight.142547)
Supplement: Supplemental data [file jciinsight-6-142547-s159.pdf]

Abbreviations: CI, confidence interval; ECOG PS, Eastern Cooperative Oncology Group Performance Status; HR, hazard ratio; PFS, progression-free survival.

Supplemental Methods
Molecular Matching Score

In general, the molecular Matching Score was calculated by dividing the number of alterations matched in each patient (numerator) by the number of characterized aberrations in that patient's tumor (denominator, several mutations in the same genes counted as one aberration; all variants of unknown significance were excluded). Detailed information on Matching Score calculations has been previously published. (28) The same formulas were followed except that herein, each altered component of the cell cycle pathway was counted separately rather than as a single unit, e.g., if a patient had both *CDK4* amplification and *CDKN2A/B* loss, they were counted separately. As an example, for calculating Matching Score, if a patient who had a cancer harboring eight genomic aberrations received two drugs that targeted four of the patient's genomic alterations, the molecular Matching Score would be 4/8 or 50%. Certain drugs that targeted more than one alteration (because small molecule inhibitors often have activity against multiple kinases) were counted as matches for each identified genomic alteration that was matched. It should be noted that, for small molecule inhibitors, matching was based on a half maximal inhibitory concentration (IC50) of the drug for the target (generally, <100 nM) or for effectors immediately downstream of the gene product altered. Antibodies were considered matched if their primary target was the product of the molecular alteration. PARP inhibitors and platinum agents were considered matched to alterations that affected BRCA-related pathways. Additional rules applied. If the patient was treated with immunotherapy, the molecular Matching Score was 100% for PD-L1 immunohistochemistry (IHC) high/intermediate positive, high tumor mutation burden (TMB), or high microsatellite instability (MSI) results. If PD-L1 IHC was low positive or the TMB was intermediate, the molecular Matching Score was 50%. The cut-off of 50% for the analyses of low versus high molecular Matching Scores was chosen according to the minimum P-value criteria (41). It should be noted that, with the use of targeted therapies, a high (≥50%) Matching Score could be achieved in the rare patient with a single alteration in the cell cycle pathway if that alteration was targeted (Matching Score = 100%) or in patients with few alterations in the

pathway, if only the cell cycle alteration was targeted; a high Matching Score was achieved in three patients in this way Otherwise, patients with multiple genomic co-alterations could only achieve a high Matching Score if a matched combination therapy was given that targeted the majority of the co-alterations along with the cell cycle gene alterations. Three of the six patients who had a high Matching Score with immune checkpoint blockade in their regimen would have had a high Matching Score even if the immune checkpoint blockade was excluded**.** No treated patient had a co-existing cell-cycle resistant alteration such as an *RB* or *CCNE1* alteration (**Supplemental Table 1)**. Matching Scores were calculated while blinded to patient outcome.

SUPPLEMENTARY INFORMATION

Supplemental Table 1. Characteristics of 40 patients with alterations in *CCND1/2/3*, *CDK4/6* and/or *CDKN2A/B* G1-S phase cell-cycle genes who received CDK4/6 inhibitor-based therapy.

Study ID	Cancer type	Tissue NGS report prior to the therapy	Regimen	Matching Score (%)	Matching Score Calculation	Line of therapy	PFS (months)
10	Lung, non-small cell	*CDK4* amplification, *MDM2* amplification	abemaciclib	≥50%	1/2=50%	1st line	6.1
64	Brain	*AKT3* amplification, *EGFR* amplification, *PIK3CG* V274I, *CDK6* amplification, *TP53* P190L, *DPP6-ETV1* potential fusion	palbociclib	<50%	1/6=17%	4th line	2
89	Brain	*EGFR* A289D, *ATM* splice site 4612-2A>C, *CDK4* amplification, *CDKN2A/B* loss, *MDM4* amplification	palbociclib	<50%	2/5=40%	6th line	0.9
102	Brain	*PDGFRA* amplification, *TP53* N131S, *CDK4* amplification	palbociclib	<50%	1/3=33%	6th line	2.8
141	GI, non-colorectal	*CDKN2A/B loss, TP53 C176F, TP53 D61fs*62, EP300 P925T*<br>Other: *TMB-intermediate (6 muts/mb)*	palbociclib/nivolumab	≥50%	50%+1/(4x2) =62.5%	6th line	4.6
202	Other (liposarcoma)	*CDK4* amplification, *MDM2* amplification, *WT1* truncation intron 3	palbociclib	<50%	1/3=33%	2nd line	8.2
220	Lung, non-small cell	*CCND3* P203S, *CDKN2A* p16INK4a R58* and p14ARF P72L, *TP53* loss exons 10-11, *RET* truncation intron 11	palbociclib	≥50%	2/4=50%	2nd line	7
231	Other (liposarcoma)	*NF1* loss exons 2-57, *STK11* F354L, *CDK4* amplification, *CDKN2A/B* loss, *MDM2* amplification, *GULP1-NOTCH1* fusion, *STAT4* truncation exon 16	palbociclib/everolimus	<50%	3/7=42.9%	2nd line	3.8
269	Gynecologic	*CDKN2A/B* loss	palbociclib	≥50%	1/1=100%	3rd line	8
302	Hepato-pancreato-biliary	*FBXW7* E113D, *CDKN2A/B* loss, *AXIN1* R395P, *TERT* promoter -124C>T	palbociclib	<50%	1/4=25%	4th line	1.1
310	Head & neck cancer	*CDKN2A/B* loss, *CYLD* S371*	palbociclib/cetuximab	≥50%	1/2=50%	6th line	5.8
315	Head & neck cancer	*PIK3CA* amplification, *PTEN* Q298*, *CDKN2A* p16INK4a D84Y and p14ARF R98L, *PRKCI* D396E, *SOX2* amplification, *TP53* Q144*, *NFE2L2* W8C	palbociclib/cetuximab	<50%	1/7=14.3%	2nd line	6.3

327	Brain	*EGFR* amplification, EGFRvIVa, *CDKN2A* loss p16INK4a and p14ARF exons 2-3, *PIK3R1* N453del, *QKI* E135fs*5, *SETD2* splice site 5016-2_5018delAGAAA, *TERT* promoter -124C>T	abemaciclib/nivolumab/ bevacizumab/ osimertinib	<50%	2/7=28.6%	4th line	0.6
334	Head & neck cancer	*BRIP1* N196S, *TSC1* S575fs*12, *CDKN2A/B* loss	palbociclib	<50%	1/3=33.3%	5th line	0.6
351	Hepato-pancreato-biliary	*AKT2 amplification, KRAS G12R, TP53 splice site 919+1G>A, CDKN2A/B loss*	palbociclib/ nab-paclitaxel	<50%	1/4=25%	3rd line	1.9
391	Gynecologic	*CDKN2A* p14ARF P72L and p16INK4a R58*, *FRS2* amp, *MDM2* amp	palbociclib/lenvatinib/anastrozole/doxorubicin	≥50%	2/3=66.7%	3rd line	6.2
394	Lung, non-small cell	*EGFR* exon 19 deletion (E746_A750del), *CTNNB1* S37F, *CDKN2A/B* loss	palbociclib/osimertinib	≥50%	2/3=66.7%	6th line	4.6
404	Other (liposarcoma)	*AKT1* amplification, *CDK4* amplification, *MDM2* amplification, *CDC73* rearrangement intron 14, *FRS2* amplification, *ZRSR2* R446_R448>R	palbociclib/lenvatinib	<50%	2/6=33.3%	1st line	3.5
416	GI, colorectal	*KRAS* G12D, *APC* K1165*, *APC* P1373fs*10, *CDKN2A* p14ARF S73R, *SOX9* Y420*, *TP53* R110L	palbociclib/trametinib/sulindac	≥50%	3/5=60%	2nd line	6.8
424	Other (chondrosarcoma)	*CDKN2A/B* loss exon 2, *SETD2* splice site 4455-2_4456delAGAA<br>Other: Progesterone-receptor positive by immunohistochemistry	palbociclib/lenvatinib/anastrozole	≥50%	2/3=66.7%	5th line	3.7+
425	Brain	*CDKN2A/B* loss , *EGFR* amplification, *EGFR* G598V , *PIK3R1* I571fs*31 , *TERT* promoter -124C>T	palbociclib/lapatinib/metformin/cetuximab/nelfinavir	≥50%	3/5=60%	2nd line	1.3
443	Hepato-pancreato-biliary	*MDM2* amplification, CDKN2A p16INK4a R80* and p14ARF P94L, *CEBPA* G103_G104del, *FRS2* amplification	palbociclib/lenvatinib	≥50%	2/4=50%	1st line	11.5
448	Hepato-pancreato-biliary	*PTCH1* V1131M, *ARID1A* R1026fs*13, *BAP1* D672G, *CDKN2A/B* loss	palbociclib/nivolumab/vismodegib/olaparib	≥50%	3/4=75%	3rd line	1.6
450	Head & neck cancer	*BRCA1* R1744*, *CDKN2A/B* loss, *SMAD4* loss exons 1-10, *TP53* E258G, *TP53* R156G, *TP53* Y220C	palbociclib/cetuximab	<50%	1/5=20%	4th line	4.5
459	GI, non-colorectal	*TSC2* splice site 2967-2A>T, *MYC* amplification, *RICTOR* amplification, *CCND3* amplification, *CTCF* rearrangement exon 11, *MYST3* amplification, *TP53* R175H, *VEGFA* amplification<br>Other: PD-L1 5% positive by IHC (tumor proportion score)	Palbociclib/nivolumab/ bevacizumab	≥50%	50%+3/(8x2)=68.8%	1st line	8

464	Head & neck cancer	*EGFR* amplification, *CCND1* amplification, *CDKN2A* p16INK4a W110* and p14ARF G125R, *SUFU* splice site 1158-1G>T, *FAT1* A1564fs*10, *FGF19* amplification, *FGF3* amplification, *FGF4* amplification, *TP53* splice site 375G>T, *TP53* splice site 376-1G>A	palbociclib/cetuximab	<50%	3/10=30%	1st line	1.8
466	GI, colorectal	*BRCA2* S3332Y, *CCND2* amplification, *STK11* loss exon 1, *CDKN2A* p16INK4a A68T and p14ARF R82H, *FGF23* amplification, *FGF6* amplification, *KDM5A* amplification, *SMAD4* loss exons 1-10, *TP53* splice site 376-1G>C	palbociclib/olaparib/bevacizumab	<50%	4/9=44.4%	4th line	0.7
474	Head & neck cancer	*CCND1* amplification, *CDKN2A/B* loss, *FGF19* amplification, *FGF3* amplification, FGF4 amplification, *KMT2C* (*MLL3*) M3463fs*32, *TP53* I255del<br>Other: PD-L1 1% positive by IHC (tumor proportion score)	palbociclib/nivolumab/ lenvatinib	≥50%	50%+6/(7x2)=92.9%	1st line	2.6
477	GI, non-colorectal	*CCND1* amplification, *FGF19* amplification, *FGF3* amplification, *FGF4* amplification, *PMS2* N371fs*2, *TP53* S241F, *TP53* splice site 993G>A	palbociclib/everolimus/ bevacizumab	<50%	2/6=33.3%	2nd line	6.8
482	Hepato-pancreato-biliary	*KRAS* Q61H, *CDKN2A/B* loss, *FAM123B* E370*, *GATA6* amplification, *GNAS* R201C, *SMAD4* R135*	palbociclib/trametinib/anakinra	≥50%	3/6=50%	2nd line	1.7
488	Gynecologic	*FLT4* amplification, *PDGFRB* amplification, *CDK6* amplification, *FGFR4* amplification, TP53 K132R	palbociclib/lenvatinib	≥50%	4/5=80%	12th line	10.8+
489	Other (carcinoma of unknown primary)	*PTEN* loss exons 1-2, *STK11* Q214*, *CDKN2A* p16INK4a I49T, *SMAD4* loss<br>Other: PD-L1 60% positive by IHC (tumor proportion score)	palbociclib/nivolumab/ temsirolimus	≥50%	50%+3/(4x2)=87.5%	1st line	10.4
490	Hepato-pancreato-biliary	*KRAS* G12D, *CDK6* amplification, *CDKN2A/B* loss, *KDM6A* V326fs*38, *TP53* S127F, *ZNF703* amplification<br>Other: PD-L1 5% positive by IHC (tumor proportion score)	palbociclib/pembrolizumab	≥50%	50%+2/(6x2)=66.7%	2nd line	0.8
501	GI, non-colorectal (GIST)	*BRAF* V600E, *CDKN2A* p16INK4a splice site 150+1G>A, *LRP1B* deletion exon 23	palbociclib/dabrafenib/trametinib	≥50%	(2+1)/(3+1)=75%	11th lline	11.3
502	Hepato-pancreato-biliary	*KRAS* G12D, *KRAS* G12R, *CDKN2A* loss exons 1-2 and *CDKN2B* loss, *SMAD4* deletion exon 11, *TP53* R267W	palbociclib/trametinib/bevacizumab	≥50%	2/3=66.7%	1st line	17.5

503	Hepato-pancreato-biliary	*KRAS* G12D, *CDKN2A* p16INK4a E27*, *GNAS* R201H, *TP53* splice site 919+2T>G	palbociclib/trametinib/bevacizumab	≥50%	4/4=100%	2nd line	2.3
504	Hepato-pancreato-biliary	*ERBB2* amplification, *KRAS* G12D, *CDKN2A* p16INK4a R80* and p14ARF P94L, *FAM123B* Q349fs*29, R*ARA-WIPF2* fusion, *TOP2A* amplification, *TP53* Q100fs*23	palbociclib/trastuzumab/lapatinib/trametinib	≥50%	(3+1)/(7+1)=50%	4th line	4
505	Hepato-pancreato-biliary	*CDKN2A* R80*, *KRAS* G12D, *SMARCB1* R201Q, *NF2* I126S	Palbociclib/trametinib/anakinra	≥50%	3/4=75%	3rd line	9.2
506	Other (osteosarcoma)	*CDK4* amplification, *CCND2* amplification, *MDM2* amplification, *FGF23* amplification, *FGF6* amplification, *FRS2* amplification	palbociclib/lenvatinib	≥50%	5/6=83.3%	3rd line	31.2
507	Other (carcinoma of unknown primary)	*EGFR* amplification, *APC* L1129S, *CDKN2A* H83Y, *PDGFRA* N33Y	palbociclib/cetuximab/erlotinib/sulindac	≥50%	(3+1)/(4+1)=80%	2nd line	4

Abbreviations: ECOG-PS, Eastern Cooperative Oncology Group Performance Status; GI, gastrointestinal cancers; IHC, immunohistochemistry; PFS, progression-free survival.
